# An RNA tertiary switch by modifying how helices are tethered

**DOI:** 10.1186/s13059-014-0425-z

**Published:** 2014-07-30

**Authors:** Laura R Ganser, Anthony M Mustoe, Hashim M Al-Hashimi

**Affiliations:** Department of Biochemistry and Chemistry, Duke University Medical Center, Durham, NC 27710 USA; Department of Biophysics, University of Michigan, Ann Arbor, MI 48109 USA

## Abstract

A viral tRNA-like structure has evolved a unique strategy to undergo a tertiary structure conformational switch that may help regulate viral regulation.

The finding that non-coding RNAs (ncRNAs) are abundant and critical players in gene expression and regulation is one of the most significant discoveries in modern molecular biology [1]. Many ncRNAs function by exploiting RNA's remarkable ability to undergo large conformational changes in response to specific biological stimuli [2]. This provides a basis for the many RNA-based molecular switches that are widely integrated into genetic circuits and biochemical pathways [2]. A recent paper by Kieft and colleagues [3] reveals a viral RNA structure that has modified the way in which its secondary structure helices are tethered together, thereby achieving a unique capacity to act as a tertiary conformational switch that may help regulate viral replication and translation.

The focus of the study by Colussi *et al.* [3] is a tRNA-like structure (TLS) located at the 3’ end of the turnip yellow mosaic virus (TYMV) genome. TYMV is a positive-sense RNA virus that contains a single-stranded RNA genome, which acts both as the mRNA template for viral protein translation by cellular host ribosomes and as the RNA template for virus replication by a viral RNA-dependent RNA polymerase (RDRP). Whereas translation proceeds from the 5’ to 3’ end of the viral RNA genome, negative-strand synthesis during replication proceeds in the opposite 3’ to 5’ direction. A regulatory mechanism is thought to prevent the two processes from occurring simultaneously, but its details remain poorly understood.

The TLS is so named because since its discovery it has been presumed to closely mimic the three-dimensional structure of tRNA. Early experiments showed that the 3’ terminus of the TLS was aminoacylated with a valine by host amino-acyl synthetases (aaRSs) [4]. This ability of the TLS to mimic tRNA structure, and thus be recognized by the host cellular tRNA machinery, allows the virus to hijack the host for its own purposes. In particular, host elongation factor proteins have been shown to bind to aminoacylated TLS and enhance translation [5]. The bound elongation factor proteins are in turn thought to deliver TLS to the host ribosomal A-site, where the 5’ end of the viral genome can loop around to bind as an mRNA template. Elongation factor binding to the TLS also inhibits negative-strand synthesis [5,6] *in vitro*, possibly by blocking RDRP binding. Importantly, the terminal 3’-CCA sequence element of the TLS also acts as a promoter for negative-strand synthesis during replication, but only when the TLS in not aminoacylated or bound to elongation factors [5,6]. It is thought that viral protein interactions with the 3’ end may promote replication and inhibit TLS aminoacylation, elongation factor protein binding, and translation from the 5’ end, but the mechanisms of how this is achieved remain poorly understood.

Early on, it was unclear whether the TLS could indeed adopt a tRNA-like conformation because it lacked many characteristic features of canonical tRNA sequences. The TLS secondary structure determined using nuclease probing data [7] subsequently revealed how the TLS achieved a tRNA-like fold and highlighted some important differences from canonical tRNA particularly at the acceptor stem, which is the site of aminoacylation [7]. In canonical tRNA, the acceptor stem is formed by base pairing of residues in the 5’ and 3’ ends of tRNA (Figure 1a). The TLS features a truncated 5’ strand that is not involved in canonical pairing with the 3’ strand (Figure 1b). This presumably is important to free up the 5’ end to link up with the rest of the viral genome without sterically obstructing the 3’ end. The longer 3’ strand was then proposed [7] to adopt a so-called ‘pseudoknot’ - an RNA motif in which a single strand folds back on itself to form two helical stems in such a way that the strand termini are located on opposite ends of the motif. This makes it possible to create an acceptor-like stem out of the 3’-terminus that can be aminoacylated (Figure 1b).

**Figure 1:**
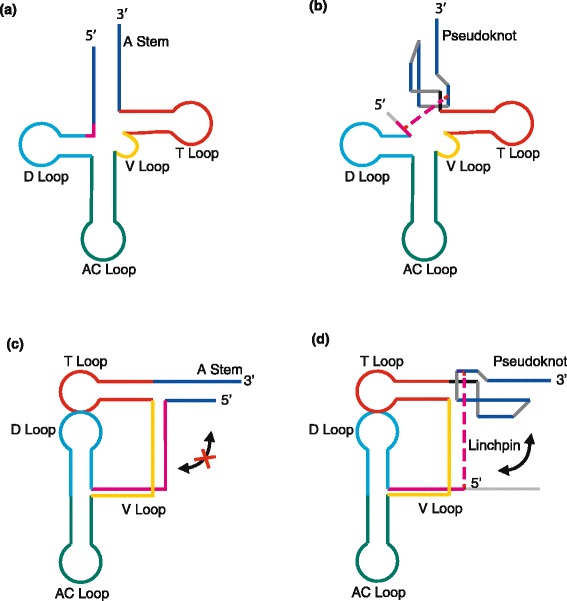
**Secondary structure of tRNA (a,c) and TYMV TLS (b,d) in cloverleaf (a,b) and L-shape (c,d) conformations.** A, acceptor; AC, anticodon; V, variable. Red lines denote the linchpin in TLS and the corresponding junction in tRNA; dotted lines represent the interactions lost following removal of the 5’-UUAG sequence. **(a)** The cloverleaf diagram shows that the junction between the D- and A-stems found in tRNA is absent in TLS, but the linchpin forms an interaction in its place. **(b)** The L-shape fold of the TLS is stabilized by the linchpin interaction. Destabilizing this interaction allows it to extend, as shown by the arrows, whereas the closed junction of tRNA prevents this extension.

Given this unique secondary structure, it remained unclear how TLS could indeed form an L-shaped conformation similar to that of a canonical tRNA (Figure 1c). Notably, the acceptor- and D-stems are no longer tethered by a two-nucleotide single-stranded connector, as is the case for canonical tRNA. A recent study [8] has shown that such ‘topological’ constraints imposed by the finite length of such connectors are critical in restricting the orientation of helical stems and in defining RNA tertiary structure. Indeed, previous studies have shown that cutting this single-stranded connector in tRNA leads to significant destabilization of tertiary structure. Moreover, many of the canonical tRNA tertiary interactions are incompatible with the TLS primary sequence and secondary structure.

Recently, work by Kieft and colleagues verified the proposed TLS secondary structure and provided compelling evidence using small-angle X-ray scattering (SAXS) that the TLS does indeed adopt an L-shaped three-dimensional structure [9,10]. However, the low resolution structural information obtained from SAXS did not reveal what interactions stabilize the TLS fold. Intriguingly, these studies also showed that TLS loses its tRNA-like L-shape upon deletion of the 5’-UUAG sequence element directly upstream of the TLS [9,10] (Figure 1d). Moreover, deleting the 5’-UUAG sequence element decreased the levels of TLS aminoacylation *in vitro* by 25 % [10]. This led Kieft and co-workers to hypothesize that disruption of interactions involving these nucleotides could cause a conformational change favoring replication over translation [9,10].

The recent high-resolution X-ray structure by Colussi *et al.* [3] confirmed the previously proposed secondary structure and shows how a tRNA-like tertiary fold is stabilized using a unique set of interactions. In particular, the guanine of the 5’-UUAG sequence element immediately upstream of the TLS base-pairs with a cytosine in the 3’ pseudoknot to form a ‘linchpin’ interaction, which is stabilized by base stacking of the neighboring 5’ adenine and V-loop residues. The linchpin effectively tethers the acceptor- and D-stems (Figure 1b). Furthermore, residues in the V-loop that normally form tertiary interactions with the D-loop in canonical tRNA form unique interactions in TLS with the linchpin and a separate upstream pseudoknot domain. The structure beautifully highlights how the same tertiary structure can be stabilized using a different topological organization and unique set of tertiary interactions.

Significantly, the structure also offers a clear explanation for why removal of the linchpin residues would destabilize the molecule, leading it to a transition from an L-shaped to a more extended conformation [9,10]. The linchpin tethers the D- and A-stems together, placing a connectivity constraint on the structure similar to that imposed by the two-nucleotide linker in canonical tRNA. Similar to the way in which breaking this two-nucleotide linker in canonical tRNA destabilizes three-dimensional structure, disrupting the linchpin interactions greatly increases the conformational freedom of the TLS stems, promoting a global loss of tertiary structure (Figure 1d).

Colussi *et al.* [3] propose that following initial infection, and once a sufficient number of RDRP proteins have been translated from the TYMV genome, RDRP binding to the TLS could destabilize the linchpin interaction, thus causing a conformational switch from the tRNA-like L-shape towards the extended TLS. This in turn would disfavor binding of aaRSs and elongation factors, providing the basis of the translation/replication regulatory switch [3]. Although there is no evidence that the two distinct TLS conformations can dynamically interchange, the linchpin interaction does seem to have been integrated into the replication promoter in order that replication efficiently disrupts the L-shaped tRNA structure required for translation.

The RNA-based conformational switches that have been characterized so far generally feature changes in secondary structure that expose or sequester key regulatory elements through formation of base-pairing interactions [2]. The TLS conformational switch reported by Colussi *et al.* [3] is a rare example of an RNA ‘tertiary switch’ that acts by modifying global three-dimensional structural features of an RNA. In a canonical tRNA secondary structure architecture, disrupting three-dimensional structure would require energetically costly disruption of multiple secondary structure helices. Altering the secondary structure connectivity and using a linchpin interaction to re-tether helices offers a potentially general strategy to construct a tertiary RNA switch. There seems to be no end to the versatility of RNA conformational switching.

## Abbreviations

aaRS: aminoacyl tRNA synthetase
RDRP: RNA-dependent RNA polymerase
TLS: TRNA like structure
TYMV: Turnip yellow mosaic virus
